# Are the ecological effects of the “worst” marine invasive species linked with scientific and media attention?

**DOI:** 10.1371/journal.pone.0215691

**Published:** 2019-04-18

**Authors:** Nathan R. Geraldi, Andrea Anton, Catherine E. Lovelock, Carlos M. Duarte

**Affiliations:** 1 Red Sea Research Center (RSRC) and Computational Biosciences Research Center (CBRC), King Abdullah University of Science and Technology (KAUST), Thuwal, Kingdom of Saudi Arabia; 2 The School of Biological Sciences, The University of Queensland, St Lucia, Queensland, Australia; University of Waikato, NEW ZEALAND

## Abstract

Non-native species are a major driver of environmental change. In this study we assessed the ecological impact of the “worst” non-native species and the associated scientific and media publications through time to understand what influences interest in these species. Ecological effect was based on a qualitative assessment reported in research publications and additional searches of the scientific and media attention were conducted to determine published articles and assess attention. We did not detect a relationship between the number of publications for a non-native species and the magnitude of the ecological effects of that species or the number of citations. Media coverage on non-native species was low, only evident for less than 50% of the non-native species assessed. Media coverage was initially related to the number of scientific publications, but was short-lived. In contrast, the attention to individual non-native species in the scientific literature was sustained through time and often continued to increase over time. Time between detection of the non-native species and the scientific/media attention were reduced with each successive introduction to a new geographic location. Tracking publications on non-native species indicated that media attention does seem to be associated with the production of scientific research while scientific attention was not related to the magnitude of the ecological effects.

## Introduction

Society’s environmental perception of non-native species is complex, and includes multiple interacting social-ecological facets, including scientific research and cultural norms [1–3]. For example, economic drivers can influence public concern over environmental issues, as demonstrated for climate change [4], and the media can shape public opinion. Given the increasing audience that scientists can reach with social media and the dialogue that exists between scientists and the public [5], the influence of science communication and its magnification by the media on societal environmental perceptions is likely to increase.

A key factor that influences human perception of non-native species is the ecological effects of these species [6], which include the positive, neutral and negative impacts on native ecosystems. People’s perception of the effects of non-native species is complicated in that there is no comprehensive list of the truly disruptive non-native species based on their ecological effects globally, even though this is mandated in Aichi Biodiversity Target 9 of the Convention on Biological Diversity of the United Nations [7]. One of the most useful references to identify the most detrimental non-native species is the IUCN’s “100 of the world’s worst invasive alien species”, which is also the Global Invasive Species Database [8,9]. This list is based on expert opinion and is widely used and cited (more than 2600 citations on Aug. 6^th^ 2018; see methods for Google Scholar search details). The non-native species included on this list are referred to as the “worst” invasive species, terminology that we will also use in this study. The attention to this prominent list, by both researchers and the media, may be influencing people’s perception of non-native species. In this paper we investigated if researcher’s and society’s perception of invasive species are influenced by the reported ecological effects of non-native species on ecosystems and the media attention given to those species.

Here we assessed the ecological effects of some of the “worst” invasive species in marine ecosystems based on Luque *et al*. (2014) and follow the scientific and media attention given to them through time, to explore how scientific research and media interest follow the trajectory of non-native species, from detection to relevant publications. Specifically, we first compared the scientific interest in non-native species among three ecosystems (marine, freshwater, and terrestrial) and then compare these to other relevant ecological research topics (biodiversity and ocean acidification) to attain a general perspective on the scientific attention given to non-native species. Second, we reviewed marine non-native species, primarily from a list of the “worst” invasive species [8,9] (S2 Table), to assess their qualitative ecological impact and compare this with the number of scientific studies and citations. We then provide summaries about their invasion histories and include a terrestrial and freshwater non-native species in this analysis for comparison. Finally, we examined the dynamics of research and media attention (based on publications) on non-native species to characterize patterns and longevity in attention.

## Methods

Broad indicators of scientific interest in invasive species were assessed by searching the Web of Science (WS) for articles that focused on the influence of non-native species in three ecosystems; marine, terrestrial and freshwater (see S1 Table for search terms and the number of references). We quantified the number of articles each year from the searches and compared the scientific interest on invasive species to other broad and relevant marine ecological topics, including effects on “biodiversity” and of “ocean acidification”, assessed through similar WS searches (S1 Table). To measure the trend through time we calculated the regression line for the articles per year, which was log transformed to compare to previous findings. The WS searches were performed on the 24^th^ of September 2018.

Attention to individual non-native species was assessed by focusing on “100 of the World’s Worst Invasive Alien Species” [8,9]. The authors indicated that the species included in this list of the worst invasive species were selected for their serious impact on biological diversity and/or human activities but advised “absence from the list does not imply that a species poses a lesser threat, and their illustration of important issues of biological invasion. To ensure a wide variety of examples, only one species from each genus was selected” [8]. This list was updated in 2014 [9], but with no change in the marine species included in the list. Lowe et al. (2000) continues to be referenced and was cited 2674 times, with 510 of these after 2016 (Google scholar search on Aug. 6^th^ 2018).

Eleven marine species were identified on the “worst” invasive species list (S2 Table; Luque et al. 2014). We also included the non-native red lionfish (*Pterois* spp.) in the analyses given their recent and rapid invasion of the mid-western Atlantic Ocean, as well as two of the “worst” invasive species from terrestrial and freshwater systems for comparison with our marine non-natives: the terrestrial cane toad (*Rhinella marina*, formally *Bufo marinus*) and the freshwater zebra mussel (*Dreissena polymorpha*; S2 Table). To determine the temporal trends in scientific and media interest on individual non-native species we searched the WS and ProQuest’s ABI/INFORM collection (ABI/INFORM). The ABI/INFORM collection is an international English-language business research database that contains thousands of full text newspapers, magazines, wire feeds, trade journals and web based information starting in the 1970s. The species-specific ABI/INFORM and WS searches were performed from the 20^th^ to the 26^th^ of June 2016. Scientific journals and dissertations were excluded from the ABI/INFORM search to minimize overlap with WS search. Of the 14 species searched, only the results of eight species with appreciable media coverage were assessed, because the other species had almost no media coverage based on our searches.

The scientific interest in these eight individual species identified and the timeline of when they were labeled non-native or invasive were determined by two separate WS searches for each species. Although we do not know the exact definitions used by authors within publications, the following definitions are commonly used within scientific literature and for this reason we did two separate searches: The term ‘non-native’ is used to refer to species that have been moved outside their native geographic ranges via human actions while the term ‘invasive’ is used for established non-native species that are spreading in the new environment (*sensu* [10] and/or have a demonstrable ecological or economic impact *sensu* [11]). Thus, invasive species are a small subgroup of non-native species that are spreading fast and/or exert a clear ecological or economic impact. The first WS search included the following structure, “invader OR non-native OR exotic OR alien NOT invasive AND scientific name”. The second search consisted of “scientific name AND invasive”. Finally, to qualify the qualitative ecological effect of the six marine invasive species, a third WS search for each species was conducted using the following terms: (invader OR non-native OR exotic OR invasive OR alien AND scientific name) NEAR/5 (impact OR effect OR influence OR consequence*) AND (marine OR coastal OR sea OR estuar* OR ocean).

The reported ecological effect of the non-native species was determined based on the following ranking from null to broad ecosystem effects (adapted from [12]: 0—No effect; 1—effects 1 species with little or no wider ecosystem impact; 2—effects multiple species, some wider ecosystem function or species of high conservation value; and 3—effects entire ecosystem processes and/or wider abiotic influences). A cumulative effect was made for each paper. Agreement among observers was checked by all observers ranking 3 of the same papers and observer ranks were identical. Only studies that included a quantitative comparison with and without the non-native species were included. To ensure the scientific and media search results were related to the focal non-native species and not another species or topics such as aquaculture or aquariums, references were individually inspected and articles not about the focal non-native species were removed (S1 and S2 Table for details). Three separate analysis were run to assess if there were relationships between focal species, attention, and ecological effects. To determine if the attention given to a species was related to the ecological effect a general linear model (glm) was run with the number of publications per species as the independent variable and the mean effect per species as the dependent variable. To assess if the number of publications per species was related to the mean number of citations a glm was run with the number of publications per species as the independent variable and the mean number of citations per year for each species as the dependent variable. Finally, to determine if there the ecological effect found by a study was related to the number of times a publication was cited, a glm was run with the ecological effect as the independent variable and the number of citations per year as the dependent variable. It is important to note that there is not a perfect procedure to standardize the number of citations for publications published in different years because the years since publication and number of citations is not linear. However, the accumulation of publication is relatively constant for the first 10 years, although the most citations often occur 2 years after publication [13]. To incorporate the decline in citations after 10 years of publication, publications that were more than 10 years old were always divided by 10 instead of their actual years since publication. All analyses were run in R version 3.5.1 [14].

To elucidate patterns between introductions and attention, we matched the location of the study for scientific articles or the location of the focal non-native species for the media article with the year of introduction obtained from the Invasive Species Compendium (CABI; www.cabi.org). Because the exact year of introduction is usually unknown our calculations are referred to as the year of detection. CABI gives a detection year by country, but includes states/provinces for larger countries (i.e. USA and Australia). The detection location from CABI was then broadened to a region to encompass the location of the article. Matching detection location with article location was checked for accuracy based on the detection, distribution and spread given in CABI. Thus, our definition of region was dependent on the detection and spread given in CABI and the geographic distributions found in articles (region needed to encompass all articles associated with each species specific introduction), which varied from state (Florida for the *Rhinella marina*) to continent (e.g. Europe for *Undaria pinnatifida*). If the focal location of article could not be identified the location was categorized as general.

The attention span, whether scientific or mediatic, to each focal non-native species was measured by focusing on the 2 separate years with the greatest references and dividing the number of references in these peak years by the number of references in the following year. Peaks with less than 5 references were not included. The lag in attention between detection and peak articles, scientific and from the media, was then quantified for each species introduction by subtracting the years with the first and most references from the region-specific detection year. The lag in attention was calculated for both the media and the two scientific searches (e.g., using either the non-native or the invasive labels). Finally, we looked at how attention changed as a non-native species was successively introduced into new regions. This was accomplished by quantifying the time between detection and first article for each species for each region. The slope of time between detection and first article for each successive introduction was determined for each reference search and differences in the slope among scientific and media articles was tested with a Kruskal-Wallis test with each species as a replicate. Data are provided in S3–S6 Tables.

## Results and discussion

### Scientific attention to invasion ecology and comparisons among ecosystems

**Fig 1:**
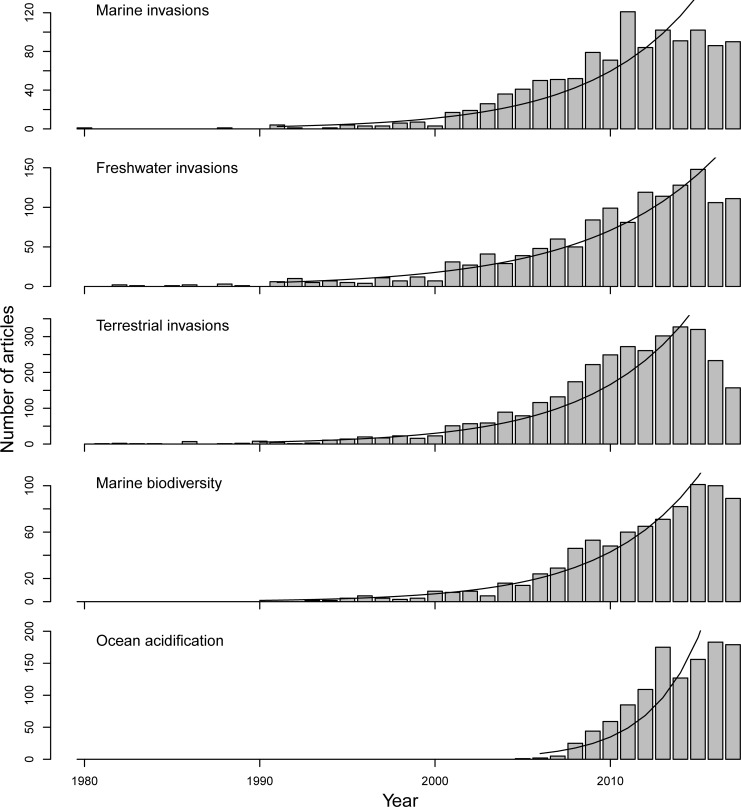
The number of scientific articles published on the effect of non-native species in marine, freshwater and terrestrial ecosystems (top 3 plots) and on biodiversity and ocean acidification in marine ecosystems. Regression lines show the trend of the log-transformed, annual number of articles.

Research attention to the effects of non-native species has grown steeply at a rate of 15.7±0.9 (SE) %yr^-1^ (Fig 1), almost three times faster than the rate of increase of general ecological literature (7.7%yr^-1^) [15]. More than 50% (56.1%) of the effort focused on terrestrial non-native species compared to 24.1% on freshwater and 19.8% on marine non-native species (Fig 1). The growth rate of research effort on the effects of non-native species has been similar for terrestrial (17.1±1.2%yr^-1^), freshwater (17.1±1.2%yr^-1^) and marine species (16.8±1.2%yr^-1^; Fig 1), but the latter increasing slightly from encompassing 14.1% of the papers published on the subject in 1995–2000 to 19.3% in 2012–2017 (Fig 1). The publication rate on the effects of non-native marine species was similar to growth in the research attention to biodiversity (18.4±0.8%yr^-1^), but less than ocean acidification (34.0±5.4%yr^-1^; Fig 1). However, the number of publications deviates from these rates in the last two to four years (2014–2017) depending on the search, indicating a leveling off or slight decrease in the number of publications per year for these topics. Compared to the 17% research effort marine non-native species received of the total research effort on non-native species (Fig 1), the list of the worst invasive species Lowe et al. (2000) includes mainly terrestrial species with only 11% of marine species (i.e. 75 terrestrial, 14 freshwater and 11 marine non-native species; S2 Table). This would suggest that future updates of this list could consider adding more marine and freshwater species to keep up with the scientific interest in evaluating the “worst” non-native species.

### Scientific attention and findings on marine non-native species

**Fig 2:**
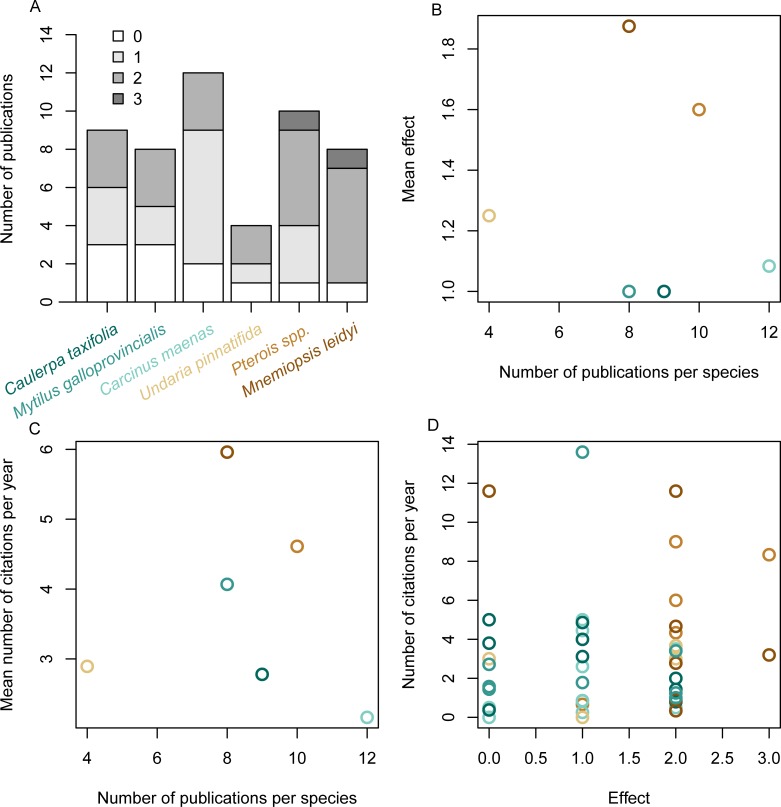
Qualitative assessment of the effects of the “worst” marine invasive species based on scientific research shown by the number of publication for each species with publications for each species being categorized by the effect (A), the number of publication for each species compared to the mean ecological effect (B) and mean number of citations per year (C) and the ecological effect compared with the number of citations per year of all publications (D). Species were ordered, from highest to lowest, by their mean effect (A) and the label color for each species indicates species data points in the other plots (C-D). The were no significant effects for the relationships shown in B-D (linear models, p-value>0.05).

Our qualitative review of the documented impacts of the “worst” non-native marine species indicates that the majority of ecological effects altered 1 species or multiple species (ranking of 1 or 2 which included 16 and 22 studies respectively). No effects were found for 11 of the 51 studies (rank of 0) and only 2 studies found an effect on the entire ecosystems (rank of 3). The mean effect of species ranged from 1 to 1.9 while the number of studies for each species ranged from 4 to 12 (Fig 2A). Given that there were less than 10 scientific publications for all but one of these species, continued research on these species is warranted to determine their long-term and region specific impacts. No relationship was detected between the scientific attention given to species (number of publications) and the mean ecological effect of the species (t-value = -1.55, df = 4, p-value = 0.884; Fig 2B). We did not detect a relationship between the scientific attention given to species (number of publications) and the number of citations (t-value = -0.2316, df = 4, p-value = 0.828; Fig 2C) or the ecological effect of each publication on the number of citations per year (t-value = 1.03, df = 54, p-value 0.306; Fig 2D). Although our results are based on only 6 species which limits the power of our analysis to detect significance, these findings suggest that scientific attention is not skewed towards species with greater ecological effects and that publications with greater effects are not cited more often than publications that found minimal effects of non-native species.

### Dynamics of attention by the media and the scientific community

**Fig 3:**
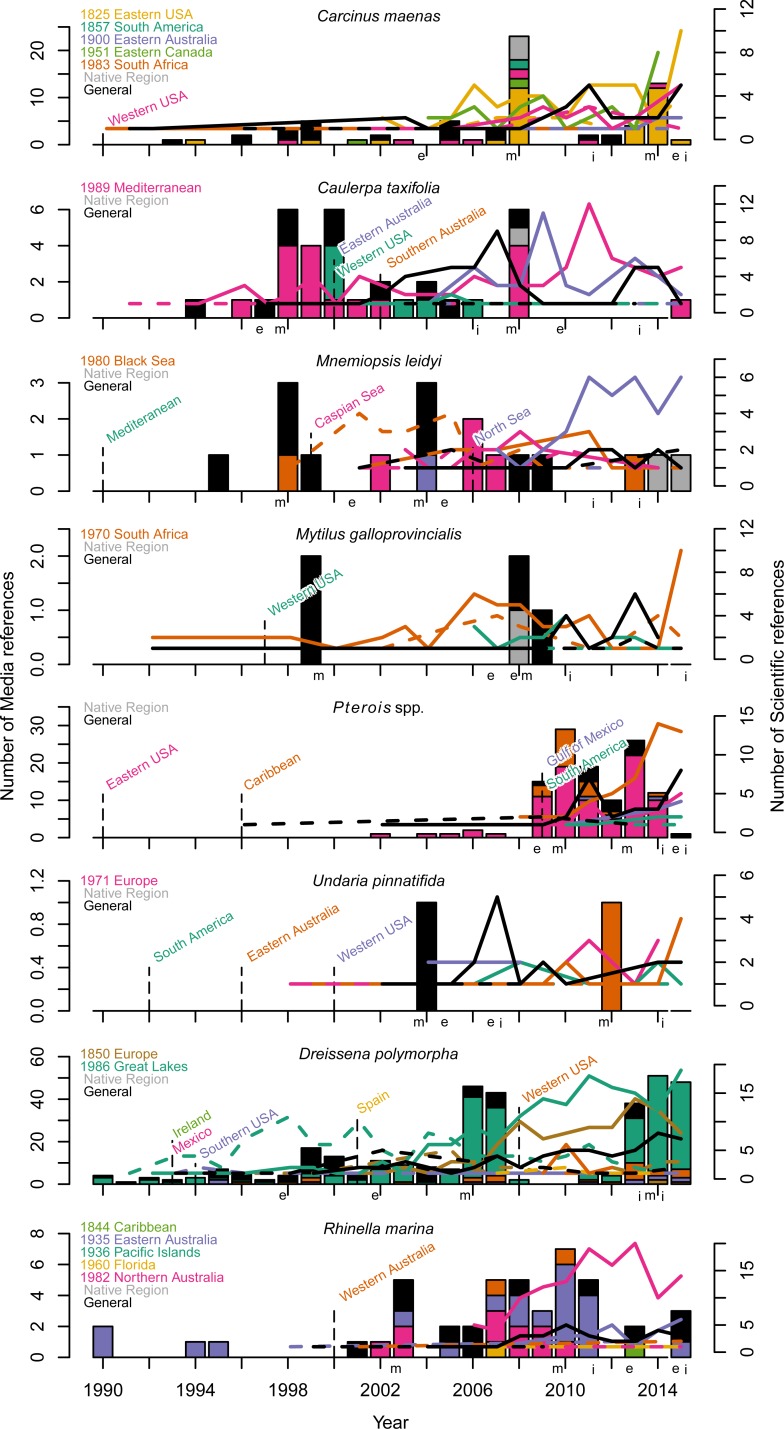
Media and scientific coverage of worst invasive species. The first six plots are marine species and the bottom two are freshwater and terrestrial species respectively. The year of detection to a certain location are shown with vertical dashed lines and labeled with region. Detection locations before 1990 are shown on the left of each plot (General indicates region not identified). Bars and left axis show the media coverage of the species. Lines and right axis show the scientific literature citing the species as non-native (dashed line) or invasive (solid line). Media and scientific references are color coded to match the color of the region to which the label non-native or invasive occurred. Letters in the x-axis indicate attention peaks: media (m), non-native (e) and invasive (i).

We observed that the number of scientific papers on a non-native species was less likely to collapse abruptly once the peak attention was reached than that of the number of references by the media (Fig 3), pointing at a shorter attention span of the media, and therefore the public, compared to scientists. For example, 4 of the 8 species examined here received the greatest scientific attention in 2015, as indicated by scientific articles on them, while no media peaks were observed after 2014 (Fig 3). The year after the peak in scientific publications, scientific attention declined by only 19% (n = 12, SE = 6%; Fig 3) for invasive species references. Media attention was in contrast short-lived and coverage of invasive species declined by 61% the year after the peak (n = 7, SE = 13%; Fig 3), i.e. three times faster than that for scientific publications. Our case studies of the lionfish in the marine environment and the zebra mussel in freshwater departed from this pattern in showing sustained media attention for the past six and three years, respectively (Fig 3). Overall, these dynamics may be affected by the different turnover of media vs. scientific publications, with media reports released almost immediately while scientific publications typically requiring more than 1 year between study completion and publication in a scientific journal [16]. Thus, media attention rises rapidly, but is short-lived, while attention to individual non-native species in the scientific literature is sustained and often continues to increase over time.

The evaluation of the regional introductions of each species indicated that there was 8.5 years (median; mean = 36.9, n = 20, SE = 10.3) from the time when a species was detected to when it was reported as non-native in that region and 11 years (median; mean = 39.1, n = 20, SE = 10.4; Fig 3) from the time when a species was detected to when it was reported as invasive in that region within the scientific literature. The number of years between the detection of the non-native species and the first media reference was 10 years (median; mean = 36.0, n = 20, SE = 10.5, Fig 3). The maximum attention given to non-native species in the scientific literature was approximately 3 and 6 years after the first non-native and invasive references, respectively. The maximum media attention came on average 6 years after the initial coverage. These results suggest that media coverage followed research publications and the shorter lag between first and maximum coverage for the scientific literature on non-native species probably results from the species being labeled invasive after publication of multiple scientific articles. The rapid translation of scientific publications on invasive species to the media (median number of years between the first scientific publication and media reference was 0.5; mean = 0.2, n = 20, SE = 1.16) highlights the contrast between the immediacy of media articles compared to the slow scientific publication process [16]. In addition, scientific articles seemed to positively correlate with media reports (Fig 3), therefore, the more scientific articles produced on a non-native species, the more media attention was given to that species.

### Subsequent introductions and species labels

**Fig 4:**
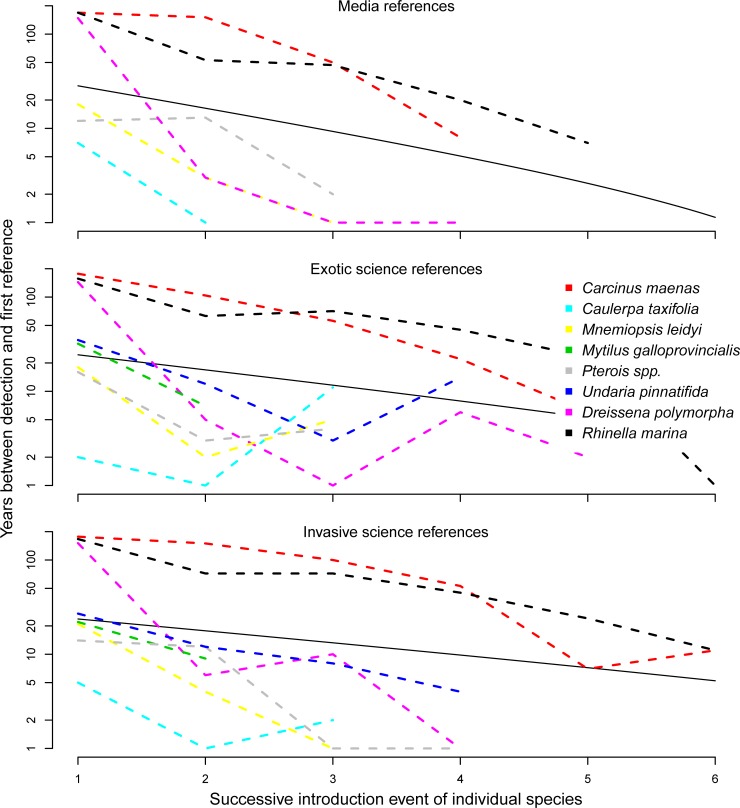
The number of years between detection and the first references for the media (top), scientific research when the species was associated with non-native (middle) and scientific research when the species was associated with invasive (bottom) in relation to chronologically ordered invaded regions for each species.

Non-native species are often introduced to multiple geographic regions, but evidence of ecological impacts in one region does not necessarily imply that the species will have similar impacts on a geographically distant region [17]. However, it is unclear how scientists and the media react to subsequent introductions. For example, is an invasive species automatically labeled invasive when introduced in a new geographic region and how does the terminology change over time with each successive report of occurrence into a new geographic region? Our analyses show that regardless of the reference type (media or scientific references), the time between detection and attention decreased each subsequent time a species was introduced into a new region (Fig 4). On average, the time between detection and the first non-native scientific reference decreased by 65±17% at each successive introduction, while the time between detection and the first invasive scientific reference decreased by 78±17% at each successive introduction (Fig 4). In addition, three of the six marine invasive species in this study (*Caulerpa taxifolia*, *Mnemiopsis leidyi and Pterois volitans*) were termed invasive within one year of the species being detected in a new geographic region. Time between detection and the first media reference quickly decreased for each successive reported presence into a new region (52±26% of the time compared to the previous introduction, Fig 4). The decreasing time between detection and first reference for each successive introduction were not statistically different among scientific attention to non-native or invasive species or media attention (differences among slopes; Kruskal-Wallis test, p-value = 0.42). Given the time it takes to undertake ecological studies and to publish in the scientific literature, it is likely that researchers were much quicker to initiate research at each subsequent introduction to determine if the species alter ecosystems in the newly introduced region. The decreased time between successive detections and attention is probably a consequence of both enhanced ability to recognize previously documented invasive species and the enhanced promotion of a species from non-native to invasive regardless of local ecological impacts.

## Conclusion

We focused on the “worst” marine non-native species to assess interactions among ecological impacts and the media and scientific attention. We did not find any indication that scientific publications or citations were associated with the magnitude of ecological effects reported in publications for individual species. These non-significant results could derive from low sample size, which in part reflects the scarce scientific and media attention given to non-native species. In general, the species were only promoted to an invasive status following scientific research on their ecological effects, and only then received media attention. However, many non-native species received little or no media attention. Once a species was labeled invasive, there was a clear trend to recognize this species and use the “invasive” label to describe that species more rapidly at each successive detection in a new geographic region.

The short-term media coverage, in general lasting from one to two years, likely results from two factors. First, the ecological impact of the invasive species loses novelty through time, and the non-natvie species are not news worthy anymore. Second, the effect of the non-native species may be low when compared to the initial predicted ecological impacts and, particularly, the exaggerated expectations of threats associated with media reports and the nicknames the species often receive (Table 1). In contrast, scientific interest was often sustained through time. Maintenance of scientific interest probably results from; 1) the precautionary principle, which necessitates robust evidence to determine the ecological effect of non-native species [18], 2) the slow publishing process in science, typically involving multiple years from funding to publication and 3) scientific interest in non-native species that goes beyond its ecological impacts and may include interest in other disciplines including evolutionary processes [19].

**Table 1:** Narratives on a few of the “worst” invasive species.

Invasive species name	Narrative of invasion
***Rhinella marina*** (Killer Cane toad)	The cane toad was introduced to Australia from South America in 1935 to help control insect pests of sugar cane [25]. Cane toads are associated with drastic declines in 3 species of predatory reptiles [26], mass mortalities of crocodiles [27] and reductions in mosquitoes [28]
***Dreissena polymorpha***	The freshwater zebra mussel, originally from rivers and lakes in western Russia and Ukraine, has received the most attention, both in the media and from scientists (Fig 2, S1 Table). The mussel has proliferated in freshwater habitats throughout North America and Europe where it covers hard substrates and interferes with infrastructure causing municipalities to invest millions of dollars in clearing pipes [29]. The introduction of the mussel has resulted in drastic ecological changes, such as the shift in ecosystem production from the water to the benthos [30].
***Carcinus maenas*** (Voracious army)	From its native range in Europe and North Africa, the European green crab has spread to the east and west coast of North America, southern Australia, South Africa, and South America (Fig 3). To date, documented impacts on native species have been mostly negative in the east and west shores of North America [31–33], but positive effects on saltmarshes, by triggering trophic cascades, in the eastern USA [34]. In South Africa, they have not spread beyond protected harbors [35] and in the eastern USA have been declining since the early 2000’s [36,37].
***Caulerpa taxifolia*** (Killer weed, Rogue algae, Alien stowaway, Aggressive alien algae, Toxic seaweed)	The green algae *C*. *taxifolia*, a native of the Caribbean Sea and Northern Australia, has been accidentally introduced into the Mediterranean, California, and Southeastern Australia. The impacts of *C*. *taxifolia* have been generally contrasting even though it was initially feared to displace the key-habitat forming seagrass *Posidonia oceanica*, cause widespread disruption of coastal food webs, and even make fish that consume this alga poisonous to humans [38]. This resulted in the most invasive scientific publications of the marine species in this study (Fig 2, S2 Table). Although *C*. *taxifolia* outcompeted endemic *Posidonia oceanica* in the Mediterranean [39], it has had no measurable contribution to the widespread decline of *Posidonia oceanica* across the Mediterranean Sea [40] or Australia [41]. *C*. *taxifolia* has gone through boom and bust stages in the Mediterranean Sea and now covers only a small fraction of the area occupied during its peak in the late 1990s [42,43].
***Mnemiopsis leidyi*** (Killer jellyfish, Jellyfish pest, Caspian Sea pest)	The ctenophore *M*. *leidyi*, a native in the western Atlantic, was accidentally introduced into the Black Sea in 1980. It was associated with the collapse of multiple fisheries [44], raising this species to invasive status in 2001 (Fig 3). However, subsequent research found that such changes in the Black Sea were primarily driven by overfishing, and with management of fishing pressure the population of *M*. *leidy* has declined [45]. Subsequent introductions in other areas (e.g. Mediterranean and North Sea) have not lead to reports of significant impacts [46,47] (Fig 2). This species was labeled invasive within a year of being introduced to the North Sea based on its potential predation on cod eggs [48], but predation on fish eggs was subsequently reported to be negligible [49].
***Mytilus galloprovincialis***	The blue mussel, originally from the North Atlantic, has been introduced in the northern Pacific and South Africa. It was elevated to an invasive species status because of its rapid spread and observations that it out-competed native species [50,51]. However, the mussels ability to reduce local diversity through competition appears to be context dependent [52–54]. Research also focused on the potential loss of diversity through hybridization with native mussels, which likewise seems to be rather limited [55].
***Pterois* spp** (Voracious intruder, Ravenous lionfish)	Lionfish (*Pterois miles/volitans*) are venomous predators from the tropical and subtropical Indo-Pacific. They were first detected in Florida in 1990, spread rapidly throughout the Caribbean Basin, and have been recently spotted in the western Mediterranean Sea [56]. Lionfish have attracted the most media attention of the marine exotic species considered in this study, even though its first introduction was less than 30 years ago [57](Fig 2, S2 Table). Lionfish consume dozens of species of small crustaceans and fish [58] and are capable of severely reducing native fish populations [57,59].
***Undaria pinnatifida*** *(Killer algae)*	The Japanese kelp, a native to the temperate Northwest Pacific, was introduced in several locations in the Atlantic Ocean and recently in California. It was promoted to invasive species because of documented occurrences in non-native ranges [60,61] and potential to reduce native seaweed diversity [62]. However, multiple studies found no significant impacts on native species [63,64] and there has been reports of benefits derived from harvesting wild stands of this economically important species [65].

In parentheses are some of the terms used when reporting the species in the media. All species are on the IUCN’s worst invasive species list, except for *Pterois* spp. The first two species are not marine (*Rhinella marina* and *Dreissena polymorpha*), but are included for comparison.

Although the time span of media attention was shorter than scientific interest, media coverage seemed to track scientific interest and was concomitant with the initial intensity of scientific publications, although it was almost non-existent for some of the “worst” marine non-native species. We concur that the amalgamation of scientific research and public perceptions, influenced through the media, exerts a strong influence on managerial actions [20], which may add fuel to the current debates surrounding invasion ecology (i.e., whether non-native species are harmful *per se*) and their management [2,5,21–23]. We illustrate the dynamics of scientific and media coverage and the ecological impacts of non-native species. The ability to look at the interaction between these factors and how they affect public opinion will be much improved as long-term datasets of social media, web searches, and impact metrics of research articles become available [24]. This data, which continues to be accumulated, can help to understand the ecological perceptions of scientists, managers, and society as a whole.

## Supporting information

S1 Table The search terms used to obtain relevant references for invasive species in three ecosystems and comparison topics in marine ecosystems. (DOCX)

S2 Table Invasive species assessed in this review with the number of references found in ABI/INFORM and WS searches. The percent of the number references in the search that were appropriate, as indicated by the numbers, are given in parentheses.
(DOCX)

S3 Table The results of the WS searches for non-native species in terrestrial, freshwater and marine systems, as well as marine biodiversity and ocean acidification. (CSV)

S4 Table The results of the WS search for individual non-native species and the ecological effect from each publication. (CSV)

S5 Table The results of the WS search for individual non-native species and the focal region of the article. (CSV)

S6 Table The results of the ABI search for individual non-native species and the focal region of the article. (CSV)
