# Supplementary material for: Are the ecological effects of the “worst” marine invasive species linked with scientific and media attention?
Source: PLoS One. 2019 Apr 18;14(4):e0215691. doi: 10.1371/journal.pone.0215691 (PMC6472817; doi:10.1371/journal.pone.0215691)
Supplement: S1 Table — (DOCX) [file pone.0215691.s001.docx]

**S1Table**. The search terms used to obtain relevant references for invasive species in three ecosystems and comparison topics in marine ecosystems.

| **Topic** | **Search terms** | **ISI citations** | **ABI citations** | **Relevant ABI citations** |
| --- | --- | --- | --- | --- |
| marine invasive species | (invader OR non-native OR invasive OR non-native OR alien) NEAR/5 (impact OR effect OR influence OR consequence*) AND (marine OR coastal OR sea OR estuar* OR ocean) NOT (lake OR stream OR freshwater OR terrestrial) | 1,152 | 645 | 40 |
| terrestrial invasive species | (invader OR non-native OR invasive OR non-native OR alien) NEAR/5 (impact OR effect OR influence OR consequence*) AND (terrestrial OR land OR soil OR island OR mountain) NOT (marine OR coastal OR sea OR estuar* OR ocean OR lake OR stream OR freshwater) | 3,256 | 1116 | 186 |
| freshwater invasive species | (invader OR non-native OR invasive OR non-native OR alien) NEAR/5 (impact OR effect OR influence OR consequence*) AND (lake OR stream OR freshwater OR river OR pond) NOT (marine OR coastal OR sea OR estuar* OR ocean OR terrestrial) | 1,399 | 976 | 172 |
| marine biodiversity | biodiversity NEAR/5 (impact OR effect OR influence OR consequence*) AND (marine OR coastal OR sea OR estuar* OR ocean) NOT (lake OR stream OR freshwater OR terrestrial) | 847 |  |  |
| ocean acidification | "ocean acidification ” NEAR/5 (impact OR effect OR influence OR consequence*) | 1150 |  |  |
