# Supplementary material for: Are the ecological effects of the “worst” marine invasive species linked with scientific and media attention?
Source: PLoS One. 2019 Apr 18;14(4):e0215691. doi: 10.1371/journal.pone.0215691 (PMC6472817; doi:10.1371/journal.pone.0215691)
Supplement: S2 Table — The percent of the number references in the search that were appropriate, as indicated by the numbers, are given in parentheses. (DOCX) [file pone.0215691.s002.docx]

**S2 Table.** Invasive species assessed in this review with the number of references found in ABI/INFORM and WS searches. The percent of the number references in the search that were appropriate, as indicated by the numbers, are given in parentheses. Blanks in the table indicate no data were collected for these species.

| **Scientific name** | **Common name** | **Ecosystem** | **IUCN 100 worst** | Media references | Scientific literature | |
| --- | --- | --- | --- | --- | --- | --- |
|  |  |  |  |  | **non-native** | **invasive** |
| *Asteria amurensis* | northern pacific seastar | Marine | Yes |  |  |  |
| *Carcinus maenas* | green crab | Marine | Yes | 126 (19.41%) | 40 (44.44%) | 143 (40.63%) |
| *Caulerpa taxifolia* | caulerpa taxifolia | Marine | Yes | 44 (16.73%) | 14 (37.84%) | 176 (42.93%) |
| *Cercopagis pengoi* | fishhook water flea | Marine/Fresh | Yes |  |  |  |
| *Dreissena polymorpha* | zebra mussel | Freshwater | Yes | 444 (18.69%) | 200 (47.96%) | 336 (38.98%) |
| *Eriocheir sinensis* | mitten crab | Marine/Fresh | Yes |  |  |  |
| *Mnemiopsis leidyi* | combjelly | Marine | Yes | 32 (35.96%) | 43 (47.25%) | 83 (45.86%) |
| *Mytilus galloprovincialis* | mediterranean mussel | Marine | Yes | 7 (1.01%) | 40 (40.40%) | 101 (40.56%) |
| *Potamocorbula amurensis* | marine clam | Marine | Yes |  |  |  |
| *Pterois* spp. | lionfish | Marine | No | 151 (18.30%) | 11 (45.83%) | 117 (49.37%) |
| *Rhinella marina* | cane toad | Terrestrial | Yes | 67 (24.91%) | 19 (45.24%) | 186 (47.09%) |
| *Salmo trutta* | brown trout | Marine/Fresh | Yes |  |  |  |
| *Spartina anglica* | common cord-grass | Marine | Yes |  |  |  |
| *Undaria pinnatifida* | wakame seaweed | Marine | Yes | 4 (1.44%) | 16 (45.71%) | 58 (41.13%) |
